# Association of *MMP-2 (–1306 C/T)* Gene Polymorphism with Predisposition to Optic Neuritis and Optic Neuritis Together with Multiple Sclerosis

**DOI:** 10.3390/medicina54020029

**Published:** 2018-05-07

**Authors:** Rasa Liutkevičienė, Alvita Vilkevičiūtė, Mantas Banevičus, Raminta Miežytė, Loresa Kriaučiūnienė

**Affiliations:** 1 Neuroscience Institute, Medical Academy, Lithuanian University of Health Sciences, Kaunas LT-50161, Lithuania; alvitavilkeviciute@gmail.com (A.V.); loresa.kriauciuniene@lsmuni.lt (L.K.); 2 Department of Ophthalmology, Medical Academy, Lithuanian University of Health Sciences, Kaunas LT-50161, Lithuania; manbtukbas@yahoo.com; 3 Faculty of Medicine, Medical Academy, Lithuanian University of Health Sciences, Kaunas LT-44307, Lithuania; raminta.miezyte@gmail.com

**Keywords:** gene polymorphism, optic neuritis, matrix metalloproteinase-2

## Abstract

*Background and objective:* Optic neuritis (ON) is characterized by painful, usually monocular vision loss with decreased visual acuity and defects of the visual field and color vision. The etiology and pathophysiology of ON is not completely clear. It is thought that a matrix metalloproteinase 2 (*MMP-2)* gene plays an essential role in this autoimmune inflammatory disease. The aim of this study was to determine the relationship between the *MMP-2 (-1306 C/T)* *rs243865* gene polymorphism and ON, and that of ON with multiple sclerosis. *Materials and methods:* Patients with ON/ON and multiple sclerosis and a control group of healthy individuals were enrolled in this study. The genotyping test of the *MMP-2 (-1306 C/T)* was carried out using a real-time polymerase chain reaction (PCR) method. *Results:* Analysis revealed that T allele at the *MMP-2* (-1306 C/T) was less frequent in the ON group compared to the control group (14.5% vs. 23.3%, *p* = 0.031), and was associated with decreased likelihood of ON development (OR = 0.566; 95% CI: 0.333-0.962; *p* = 0.036). No significant associations were revealed while comparing the subgroups of ON patients with and without multiple sclerosis. *Conclusion:* The *MMP-2* (-1306 C/T) gene polymorphism was found to be associated with ON development.

## 1. Introduction

Optic neuritis (ON) is described by painful, mostly unilateral visual acuity loss, changes in visual field, and reduced color contrast sensitivity (blue-yellow in the acute period, and red-green in sub-acute period) [1]. ON is closely linked to multiple sclerosis (MS), and two-thirds of the patients diagnosed with MS can develop ON [1]. MS more often occurs among young and middle-aged people [2], with an average age of onset of 30 years; it has been reported that females are more likely to suffer than males (2:1) [3]. ON mostly affects people aged more than 50 years [4], and the ratio of men to women is 3.5:1 [5]. It is thought that a loss of signal transmission in some axons due to conduction block or ganglion cell death is the main pathogenic factor of the disease [6]. This is because of a certain inflammation process results in a delayed type IV hypersensitivity reaction. The reaction is caused by the release of cytokines and other inflammatory mediators from activated peripheral T-cells, which can cross the blood brain barrier (BBB) and cause the destruction of myelin, neural cell death, and axonal degeneration [7]. It is assumed that the BBB disruption begins the disease process in ON, while the entry of T lymphocytes and inflammatory mediators into the central nervous system is mediated by matrix metalloproteinases (MMPs) [8,9,10].

MMPs belong to the family of zinc-dependent endopeptidases which play an important role in the process of degradation of the extracellular matrix and basement membrane (BM), in relation to tumor invasiveness, metastasis, and angiogenesis [11,12,13,14,15,16,17]. MMPs production might be induced by many factors, such as cytokines, growth factors, cell-extracellular matrix, and cell-cell interaction or physical stress [18].

MMP-2 is a member of the MMP family and possesses an ability to degrade type IV collagen, which is the core component constituting the BM [12,14]. Previous studies have shown that MMP-2 is involved in carcinogenesis [19,20]. MMP-2 is detected in optic nerve head astrocytes and retina, possibly influencing optic nerve changes and demyelination; it could be important in development of autoimmune inflammatory diseases such as ON [21,22].

*MMP* polymorphisms resulting from nucleotide changes due to insertions, substitutions, or microsatellite instability within the promoter region have been identified [23]. The study by Price et al. [24] found a single-nucleotide polymorphism (SNP) in the promoter region of the *MMP2* gene (−1306 C/T; rs243865). The authors concluded that the C→T transition at nucleotide –1306, located in a core recognition sequence of stimulating protein 1 (Sp1) promoter site (CCACC box), abolished the Sp1-binding site and consequently reduced promoter activity [24]. Moreover, Vasku et al. reported a newly identified nucleotide C to T transition located at nucleotide −735 in the promoter region of *MMP-2* [25].

To our knowledge, two studies have investigated associations between the *MMP-2* rs243865 gene polymorphism and ON development in the presence or absence of MS; therefore, our aim was to determine such associations.

## 2. Materials and Methods

The study was carried out in the Department of Ophthalmology, Hospital of Lithuanian University of Health Sciences, and the Neuroscience Institute, Lithuanian University of Health Sciences. Kaunas Regional Biomedical Research Ethics Committee approved the study (No. BE-2-13, issued on 19 February 2008).

The study population comprised 62 subjects with a diagnosis of ON, and 318 in the control group. For further analysis, the patients with ON were divided into two groups: those with MS (*n* = 26) and those without (*n* = 36). All patients with an attack of ON who were admitted to the Department of Ophthalmology, Hospital of Lithuanian University of Health Sciences, between 1 January 2012, and 1 February 2018, were enrolled in our study.

Subjects with ON were included according to the inclusion/diagnostic criteria (Table 1) [1,4,26,27].

Patients were excluded if they had other diseases of the optic nerve, systemic illnesses (diabetes mellitus, oncological diseases, systemic tissue disorders, chronic infectious diseases, conditions after organ or tissue transplantation), obscuration of the eye optic system, or because of poor fundus photography quality.

Diagnosis of MS was based on the neurologist consultation and MRI records. Neurological diagnosis of MS was established according to the revised and widely accepted McDonald criteria (Table 2) [28].

The control group was created from healthy subjects who were admitted to the Department of Ophthalmology, Hospital of Lithuanian University of Health Sciences, for preventive ophthalmological examinations, taking into consideration the age and gender of patients with ON. Subjects were included in the control group if they had no ophthalmological eye disorders during ophthalmological examination, and agreed to give informed consent. The exclusion criteria were any eye disorders, and/or use of epileptic and sedative drugs.

Deoxyribonucleic acid (DNA) was extracted from the venous blood of patients. DNA was purified from the peripheral blood using the Genomic DNA Purification Kit (Thermo Fisher Scientific), or the silica gel column method, using the genomic DNA extraction kit SorpoClean™ (SORPO Diagnostics), according to the manufacturer’s recommendations.

The MMP-2 (-1306 C/T; rs243865) SNP was genotyped by the real-time polymerase chain reaction (RT-PCR) method, using Applied Biosystems (Foster City, CA, USA) allelic discrimination assay with a HT 7900 real-time PCR quantification system (Applied Biosystems, USA).

RT-PCR reagents (2X Maxima™ Probe/ROX qPCR Master mix buffer, fluorescent dye labeled markers, sterile ddH_2_O) were used to prepare an appropriate RT-PCR mixture for *MMP-2 (-1306 C/T)* determination. PCR reaction mixture (9 μL) was poured into each well of 96-well PCR plates, and then 1 μL of DNA of the samples, as well as 1 μL of negative control were added. Thermal cycling conditions for RT-PCR were as follows: denaturing at 95 °C for 10 min, followed by 45 cycles of 92 °C for 15 s and 60 °C for 90 s.

### Statistical Analysis

Statistical analysis was performed using the SPSS/W 20.0 software (Statistical Package for the Social Sciences for Windows, Inc., Chicago, IL, USA). The distribution of *MMP-2 (-1306 C/T)* genotypes in patients and control groups was evaluated for consistency with the Hardy-Weinberg equilibrium (HWE), using the χ^2^ test. The data are presented as median and interquartile range (IQR), the counts and frequencies (in percentage) are presented in Table 3. The distributions of the genotypes and alleles of *MMP-2 (-1306 C/T)* in the ON and control groups were compared using the χ^2^ or Fisher exact tests, and the association of *MMP-2 (-1306 C/T)* and ON development was evaluated using binomial logistic regression analysis. Five genetic models, including co dominant (CT vs. AA and TT vs. CC), dominant (CT + TT vs. CC), recessive (TT vs. CT + CC), overdominant (CT vs. CC + TT), and additive (T vs. C) were used to estimate this association. Odds ratios (OR) and 95% confidence intervals are reported. The selection of the best genetic model was based on the Akaike Information Criterion (AIC); therefore, the best genetic models were those with the lowest AIC values. Differences were considered statistically significant when *p* < 0.05.

## 3. Results

Characteristics of the study population are shown in Table 3. Patients with ON and control subjects were gender- and age-matched.

Table 4 shows the results of genotyping of *MMP-2 (-1306 C/T)* in patients with ON, and those of the control subjects. The distribution of the analyzed SNP genotype and allele frequencies in patients with ON and in the control group matched the HWE (Table 4). The *MMP-2 (-1306 C/T)* gene polymorphism analysis in the overall group revealed that T allele was less frequent in the ON group compared to the control group (14.5 % vs. 23.3 %, *p* = 0.031) (Table 4).

Binomial logistic regression analysis in the patients with ON and control groups was performed (Table 5). This analysis revealed that each T allele was associated with decreased porbability of ON development (OR = 0.566; 95% CI = 0.333-0.962; *p* = 0.036) (Table 5).

The genotypes of *MMP-2 (-1306 C/T)* were also analyzed in the subgroups of ON patients with and without MS (Table 6). The *MMP-2 (-1306 C/T*) gene polymorphism analysis of both ON patient subgroups did not reveal any differences in the genotype distribution between ON without MS and ON with MS. Moreover, there were no statistically significant differences in the genotype and allele distribution between the ON with MS and control groups, as well as between the ON without MS and control groups (Table 6).

Binomial logistic regression analysis in patients with ON and manifestation of MS and in the control group was performed as well. There were no statistically significant variables (Supplementary Materials Table S1).

## 4. Discussion

The impact of the *MMP-2 rs243865* gene polymorphism on the development of ON, and on ON with MS was analyzed in our study. To our knowledge, no studies analyzing the impact of the *MMP-2* (-1306 C/T) gene polymorphism on the development of ON, and on ON with MS, have been carried out. Previous studies have analyzed the *MMP-2* -1575 G/A gene polymorphism and drawn attention to the relationship between ON and MS.

It was found that MMP-9 fine-tune, and thereby promote neuroinflammatory processes [31], and that the *rs243865* gene polymorphism was associated with various pathological processes which lead to inflammation affecting the optic nerve [32]. While MMP-9 is mostly upregulated in inflammatory terms, MMP-2 is constitutively expressed in the brain [33]. Brain inflammation is initiated and sustained by lymphocyte migration across the BBB [34]. Animal models proved that MMP-2 and MMP-9 are the key elements in the induction of neuro-inflammatory symptoms, and MMP-9 activity can be considered as a reliable marker of leukocyte penetration of the BBB [35]. In addition, MMP-2 and MMP-9 are expressed in the central nervous system and have several functions. Scientific research has shown that MMPs may proteolyze the cerebrovascular BM and tight junction proteins, which could compromise vascular integrity, leading to barrier leakage and extravasation [36,37,38,39]. Other studies analyzing patients with MS found increased protein levels of MMP-2 [40,41,42], and increased in lesioned MS tissue. MMP-2 has been detected in microglial nodules and microglial-like cells, where it contributes to inflammation, and is implicated in further break down of myelin basic protein (MBP) and oligodendrocyte death [43], and destabilizes the BBB [44,45]. Aksoy et al. analyzed the *MMP-2* (-1306) gene polymorphism in patients with relapsing remitting MS, and found statistically significant differences in the frequency of CT and TT genotypes and T allele in comparing patients with relapsing remitting MS to control subjects [46]. Meanwhile, in our study, differences in the genotype and allele distribution did not show any statistical significance between ON with MS and control groups. Gašparović et al. proved that the *MMP-2* (-1575 G/A) led to a 5-year-earlier disease onset in MS patients with ON as a first symptom. However, no associations between the *MMP-9* (-1562 C/T) gene polymorphism and the disease have been proven [9].

Previous studies that investigated the interrelationship between inflammation biomarkers and neurodegeneration in the cerebrospinal fluid of ON patients have identified two different inflammatory processes during ON. One of them, leukocyte infiltration represented by chemokine (C-X-C motif) ligand 13 (CXCL13), CXCL10 and MMP-9, is possibly associated with future risk of MS, while the other, represented by osteopontin and chitinase-3-like protein 1 (CHI3L1), suggests tissue damage-related inflammation [47]. Others found no correlation between exacerbation or remission of ON and cerebrospinal fluid oligoclonal IgG bands [48]. Other reports have explored gene expression profiles of peripheral blood mononuclear cells subpopulations in the early phase of acute ON. They found that CD 19^+^ cells play a significant role in acute ON pathogenesis, and represent a possible target for immunomodulation [49].

Our study shows that ON was present as the first symptom of MS development in approximately 42% of ON patients who subsequently developed MS. ON and MS have a very similar incidence, worldwide distribution and human leukocyte antigen (HLA) associations [32]. The lesions of ON are identical to those seen in MS [50]. A study was held to compare childhood and adult ON, which revealed that approximately half of adult ON patients will go on to develop MS. However, it is also apparent that others do not [51]. According to studies performed in several countries [32,50,52,53,54] an increased frequency of the MS-associated HLA-DR15 haplotype has been demonstrated. The other study was performed in Wisconsin where 18 patients with intermediate uveitis were identified as having a significant positive association (72%) with HLA-DR15. Four of those patients were diagnosed with coexisting MS or ON, one patient with coexisting narcolepsy, and three patients with a family history of MS: this shows that HLA-DR15 may be a common predisposing factor, not only for uveitis, but for ON or MS development as well [55].

Our study has several limitations. These results need to be replicated in future studies with larger sample sizes to confirm the association between ON and the *MMP-2 (-1306 C/T)* gene polymorphism. However, we worked with very small numbers of patients according to the prevalence of ON. During the period between 1 January 2012, and 1 February 2018, all patients presenting with first attack acute ON were included in our research, so it would take a longer period to collect more patients.

## 5. Conclusions

The *MMP-2rs243865* gene polymorphism was found to be associated with the development of optic neuritis.

## Figures and Tables

**Table 1:** Optic neuritis (ON) inclusion/diagnostic criteria.

Symptoms	Typical ON
Age	Young patient <50 years
Visual acuity loss time	Acute/subacute visual acuity loss
Visual acuity loss progression	Visual acuity loss progressing for few days or few weeks
Damage	Mostly one eye
Visual acuity	↓ in 90% of cases
Visual field	Changes noticed in 97% of cases
Color vision	In acute period, blue-yellow color vision loss; in subacute period, red-green color vision loss
Visual evoked potentials (VEP)	↓ VEP latency
Optical coherent tomography (OCT)	Optic nerve disc edema (mostly in superior and nasal quadrants), noticed in 20% of patients
Pain	Acute painful visual acuity loss, especially ↑ with eye movement
Optic nerve disc	Mostly normal optic nerve disc
Vitreous	Normal
Orbit	Normal
Anamnesis	ON in anamnesis or MS in anamnesis. Patients without MS had MS-like lesions but were not followed up after ON treatment in our study, only redirected for neurological follow-up.
Neurological symptoms	Neurological symptoms, allowing to suspect MS
Treatment effect using steroids	Shortens the duration of the disease
Improvement	Spontaneous improvement in 2–3 weeks
Prognosis	Mostly good
Recurrence (5–10 years)	28%

**Table 2:** McDonald MRI Criteria for Demonstration of DIS

DIS Can Be Demonstrated by ≥1 T2 Lesion ^a^ in at Least 2 of 4 Areas of the CNS:
Periventricular
Juxtacortical
Infratentorial
Spinal cord ^b^

Based on Swanton et al., 2006, 2007 [29,30]. ^a^ gadolinium enhancement of lesions is not required. ^b^ if a subject has a brainstem or spinal cord syndrome, the symptomatic lesions are excluded from the criteria and do not attribute to the lesion count. MRI-magnetic resonance imaging; DIS-lesion dissemination in space; CNS-central nervous system.

**Table 3:** Characteristics of patients with optic neuritis (ON) and control subjects

Characteristic	ON Group *n* = 62	Control Group *n* = 318	*p*
Gender, *n* (%)			
Males	21 (31.7)	113 (35.5)	0.462 *
Females	41 (66.1)	205 (33.9)	
Age, median (IQR), years	34 (17)	36 (16)	0.294 **
With MS, *n* (%)	26 (41.9)	NA	…
Without MS, *n* (%)	36 (58.1)	NA	…
Changes in visual evoked potential (VEP), *n*	62	NA	…
Pain, *n*	62	NA	…
Exophthalmus, *n*	1	NA	…
Decreased visual acuity, *n*	62	NA	…
Laterality, *n*			
Unilateral	61	NA	…
Bilateral	1		
Decreased color vision, *n*	62	NA	…
Treatment with intravenous solumedrol and later per oral prednisolone, *n*	62	NA	…
Visual acuity (affected eye), median (IQR)			
Before treatment	0.2 (0.58)	NA	<0.001 ***
After treatment	0.8 (0.70)	NA	
Farnsworth-Munsell 100 hue test score, median (IQR)	90 (29.50)	57 (31.50)	0.004 ****
Optic nerve disc appearance			
Normal	57	NA	…
Swelling	5		
Retinal nerve fiber layer thickness, median (IQR), μM			
Superior	146 (55.5)	130.5 (14.0)	0.058 **
Temporal	65 (36.0)	67 (14)	0.762 **
Inferior	144 (61.0)	137 (21.25)	0.165 **
Nasal	91 (60.0)	90 (19.0)	0.323 **
C-reactive protein, mg/L	<4	NA	…

* Pearson’s *χ*^2^ test; ** Mann Whitney U test; *** Wilcoxon Signed Ranks test; before treatment vs. after treatment; **** Mann Whitney U test (control group—50 ophthalmologically healthy volunteers). NA, not applicable; IQR, interquartile range. Ellipses indicate *p* value not computed.

**Table 4:** Frequency of *MMP-2 (-1306 C/T*) genotype in patients with optic neuritis (ON) and control group.

Gene Marker	Genotype/Allele	Control Group *n* (%) (n = 318)	*p* HWE	ON Group *n* (%) (*n* = 62)	*p* HWE	*p* Value
*MMP-2(-1306 C/T)*rs243865	Genotype					
	C/C	190 (59.75)	0.382	44 (71.00)	0.181	χ^2^ = 5.340
	C/T	108 (33.96)		18 (29.00)		*p* = 0.069
	T/T	20 (6.29)		0 (0.00)		
	Total	318 (100.00)		62 (100)		
	Allele					
	C	488 (76.70)		106 (85.50)		0.031
	T	148 (23.30)		18 (14.50)		

MMP—matrix metalloproteinase, *p* value—significance level (alfa = 0.05), *p*-value HWE—significance level (alfa = 0.05) by Hardy-Weinberg equilibrium.

**Table 5:** Binomial logistic regression analysis in patients with optic neuritis (ON) and control group

Model	Genotype/Allele	OR (95% CI)	*p*	AIC
Co dominant	C/C	1		333.562
	C/T	0.720 (0.396–1.308)	0.280	
	T/T	0	0.998	
Dominant	C/T + T/T vs. C/C	0.607 (0.336–1.098)	0.099	337.252
Recessive	T/T vs. C/C + C/T	0	0.998	332.763
Overdominant	C/T vs. T/T + C/C	0.648 (0.305–1.375)	0.259	339.521
Additive	T	0.566 (0.333–0.962)	0.036	335.184

OR—odd ratio, CI—confidence interval, *p* value—significance level (alfa = 0.05), AIC—Akaike Information Criterion.

**Table 6:** Frequency of *MMP-2 (-1306 C/T)* genotype in patients with optic neuritis (ON) without multiple sclerosis (MS), patients with optic neuritis (ON), and multiple sclerosis (MS), and control groups.

Gene Marker	Genotype/Allele	Control Group *n* (%)(*n* = 318)	ON Group without MS (%)(*n* = 36)	*p*	Control Group *n* (%) (*n* = 318)	ON Group with MS*n* (%) (*n* = 26)	*p*
*MMP-2*(-1306 C/T)rs243865	Genotype	190 (59.75)			190 (59.75)	19 (73.10)	
	C/C	108 (33.96)	25 (69.40)	0.23	108 (33.96)	7 (26.90)	0.26
	C/T	20 (6.29)	11 (30.60)	7	20 (6.29)	0 (0.00)	0
	T/T	318 (100.00)	0 (0.00)		318 (100.00)	26 (100.00)	
	Total		36 (100.00)				
	Allele	488 (76.70)			488 (76.70)	45 (86.54)	
	C	148 (23.30)	61 (84.70)	0.12	148 (23.30)	7 (13.46)	0.10
	T		11 (15.30)	3			4

MMP—matrix metalloproteinase, *p* value—significance level (alfa = 0.05), *p*-value HWE—significance level (alfa = 0.05) by Hardy-Weinberg equilibrium.

## Supplementary Materials

The following are available online at http://www.mdpi.com/1648-9144/54/2/29/s1, Table S1: Binomial logistic regression analysis in patients with optic neuritis (ON) and in the control group.
