# Supplementary material for: Association of MMP-2 (–1306 C/T) Gene Polymorphism with Predisposition to Optic Neuritis and Optic Neuritis Together with Multiple Sclerosis
Source: Medicina (Kaunas). 2018 May 7;54(2):29. doi: 10.3390/medicina54020029 (PMC6037261; doi:10.3390/medicina54020029)
Supplement: Supplementary file 1 [file medicina-54-00029-s001.pdf]

Supplementary material

Table S1. Binomial logistic regression analysis in patients with optic neuritis (ON) and in the control group.

Model	Genotype/allele	OR (CI 95%)	P value	AIC
ON without MS				
	C/C	1		
Co-dominant	C/T	0.774 (0.367-1.635)	0.502	231.899
	T/T	0	0.998	
Dominant	T/C+T/T vs. C/C	0.653 (0.310-1.374)	0.262	233.472
Recessive	T/T vs. C/C+C/T	0	0.998	230.361
Over-dominant	T/C vs. T/T+C/C	0.856 (0.406-1.804)	0.682	234.614
Additive	T	0.603 (0.090-0.962)	0.134	232.293
ON with MS				
	C/C	1		
Co-dominant	C/T	0.648 (0.264-1.591)	0.344	184.289
	T/T	0	0.998	
Dominant	T/C+T/T vs. CC	0.547 (0.223-1.339)	0.186	184.400
Recessive	T/T vs. C/C+C/T	0	0.998	183.033
Over-dominant	T/C vs. T/T+C/C	0.716 (0.292-1.757)	0.466	185.722
Additive	T	0.524 (0.233-1.176)	0.117	183.429

MS-multiple sclerosis, ON-optic neuritis, OR-odd ratio, CI-confidence interval, p value – significance level (alfa=0.05), AIC- Akaike Information Criterion.
